# Epigenetic Programming of Synthesis, Release, and/or Receptor Expression of Common Mediators Participating in the Risk/Resilience for Comorbid Stress-Related Disorders and Coronary Artery Disease

**DOI:** 10.3390/ijms19041224

**Published:** 2018-04-18

**Authors:** Carlos Manuel Zapata-Martín del Campo, Martín Martínez-Rosas, Verónica Guarner-Lans

**Affiliations:** Department of Psychiatry, Instituto Nacional de Cardiología “Ignacio Chávez”, Ciudad de México 14080, Mexico; carlos.zapata@cardiologia.org.mx (C.M.Z.-M.d.C.); martin.martinez@cardiologia.org.mx (M.M.-R.)

**Keywords:** stress related disorder, anxiety, cardiovascular disease, coronary artery disease, programming, reprogramming, epigenetics, chemical mediators

## Abstract

Corticotrophin releasing factor, vasopressin, oxytocin, natriuretic hormones, angiotensin, neuregulins, some purinergic substances, and some cytokines contribute to the long-term modulation and restructuring of cardiovascular regulation networks and, at the same time, have relevance in situations of comorbid abnormal stress responses. The synthesis, release, and receptor expression of these mediators seem to be under epigenetic control since early stages of life, possibly underlying the comorbidity to coronary artery disease (CAD) and stress-related disorders (SRD). The exposure to environmental conditions, such as stress, during critical periods in early life may cause epigenetic programming modifying the development of pathways that lead to stable and long-lasting alterations in the functioning of these mediators during adulthood, determining the risk of or resilience to CAD and SRD. However, in contrast to genetic information, epigenetic marks may be dynamically altered throughout the lifespan. Therefore, epigenetics may be reprogrammed if the individual accepts the challenge to undertake changes in their lifestyle. Alternatively, epigenetics may remain fixed and/or even be inherited in the next generation. In this paper, we analyze some of the common neuroendocrine functions of these mediators in CAD and SRD and summarize the evidence indicating that they are under early programming to put forward the theoretical hypothesis that the comorbidity of these diseases might be epigenetically programmed and modified over the lifespan of the individual.

## 1. Introduction

The interest in the relation between the heart and the emotions comes from ancient times. Aristotle mistakenly thought that the brain was an organ that cooled down the passions from the heart; however, he also noted that negative emotions adversely impacted upon the health/disease processes [1]. In 1628, William Harvey signaled the relation between the mind and the heart [2] and in 1910, William Osler was the first researcher to publish a paper on the repercussion that emotions have upon the heart, mentioning the influence of anxiety on Angina Pectoris [3]. One of the first scientific studies on the subject was the one published by Malzberg in 1937, in which he found a six-fold increase in the mortality rate adjusted per age in patients having coronary artery disease (CAD) and involutive melancholy when compared to the general population, and 40% of deaths were attributed to heart diseases [4].

Cardiovascular (CV) diseases are the main cause of premature death and chronic disability in the world [5] and mental and psychotropic substance abuse diseases are the main causes of years lived with disability worldwide. Stress-related disorders (SRDs) cause 1.1% of the disability-adjusted life years out of those caused by all other diseases worldwide [6]. Furthermore, SRDs correspond to 10.4% of all mental and substance abuse diseases [6]. The association between neuropsychiatric and CV diseases is bidirectional; neuropsychiatric symptoms are commonly present in patients with chronic systemic diseases [7] and different types of psychiatric disorders including SRD, depression, emotional incontinence, delusions, and hallucinations, which are frequently observed after the occurrence of CAD [7,8]. On the other hand, SRDs are considered as independent incidental risk factors to develop CAD, probably triggering the disease [9]. SRD entities which have been related to CAD include generalized anxiety [10,11], panic disorder [12,13], and post-traumatic stress disorder [14], among others. The association seems to be more prevalent in men than in women [15] and approximately 36% of patients with CAD have at least one SRD. In addition, more than 45% of patients with CAD have previously had one or more SRDs in their lifetime [16]. Due to this association, it has recently been proposed that there could be a common origin to these pathological entities.

The common origin of risk/resilience to CAD and SRD could involve: (a) common anatomical brain structures participating in CV and behavioral responses; (b) common mechanisms at subcellular structures (mitochondria, endoplasmic reticulum stress, telomere length) which are regulated by reactive oxygen species (ROS), inflammation, and intracellular calcium in myocytes and neural cells; (c) microbiome effects on the brain and the CV systems; and (d) common chemical mediators. Most of these mechanisms shall be dealt with in future reviews, while the participation of common neuro-immune-hormonal mediators constitutes the aim of the present review.

Epigenetic programming of the synthesis, release, and receptor expression of common mediators participating in the alternative risk of or resilience to comorbidity to CAD and SRD since early life stages, might contribute to the long-term modulation and restructuring of CV regulation networks and, at the same time, have relevance in situations of comorbid abnormal responses to stress. The term epigenetic refers to heritable traits of the expression of genes and to the subsequent phenotypic changes without there being alterations in the DNA sequence [17,18]. The term was proposed in 1942 by C.R. Waddington [19] and the concept has been modified accompanying molecular biology advances. The structure of DNA was still a mystery at that time, but the idea that genes produced a determined phenotype based on interactions with their environment held a great deal of truth.

Epigenetic programming refers to the influence produced by the exposure to specific conditions during critical periods of early life that modify the developmental pathways of the organism, leading to stable and long-lasting alterations that have effects when the individual reaches adulthood [20]. The language of the epigenetic program is nowadays based on specific covalent modifications of the DNA and chromatin [21]. Epigenetic “readers, writers, and erasers” may be targeted by nongenomic signaling, causing re programming [22]. It is now known that epigenetic changes imply post-translational modifications of histones, the methylation of DNA, and the regulation by microRNAs. Several enzymes play a role in these modifications, among which histone-modifying enzymes such as histone acetyltransferases (HAT), which acetylate histone tails and histone deacetylases (HDAC) that deacetylate histone tails [23], can be mentioned. Another group of important enzymes comprises the histone methyltransferases (HMT) and the histone demethylases. These post translational modifications of common mediators of CAD and SRD might participate in the programming or reprogramming of their neuro hormonal release and action determining risk or resilience.

In 2002, Barker et al. [24] suggested that environmental events happening during pregnancy could have consequences in the adult life of the product, leading to diseases such as cardiometabolic diseases (CMD) or type II diabetes. When nutrients are scarce, the fetus shows developmental plasticity and is capable of preserving the brain and heart blood perfusion in detriment of the growth of other organs [20,25]. Therefore, the functioning of the brain and CV system is subject to epigenetic regulation. Post in 1999 [26] published one of the first studies that proposed that CV diseases were mediated by epigenetic marks such as DNA methylation in cytosine-phosphate-guanine (CpG) islands in the estrogen receptor gene-α in patients undergoing coronary atherectomy or carotid endarterectomy. Low birthweight, an indication of an adverse gestational environment, has been associated with high cortisol levels, activation of the hypothalamus-hypophysis adrenal (HHA) axis, increased cortisol responses to stimulation with adrenocorticotrophic hormone (ACTH), lack of habituation to stress, and increased cortisol response to psychosocial stress in adult humans [27].

Epigenetic regulation plays an important role in the determination of the adaptive or mal adaptive neural functioning and the behavioral responses to environmental stressors and maternal mental disorders may alter fetal development [28]. The mechanisms involved include maternal hormonal influences such as high levels of glucocorticoids, alterations in placental functioning and perfusion, and epigenetic mechanisms in the product [20]. There are reports of the epigenetic programming of metabolic diseases such as obesity, metabolic syndrome, diabetes mellitus, and their CV consequences [29,30]. There are also recent reports of the programming of psychiatric disorders [31]. However, there are no reports on the epigenetics of comorbidity between these entities. In contrast to the genome, which is only modified by the environment through irreversible mutations, the epigenome can be modified throughout life by active changes adopted by the individuals at different developmental stages. If changes are not induced by the individual, epigenetic programming persists and can even be inherited by the next generation. Epigenetic modifications are thus both stable and flexible [32,33]. Therefore, comorbidity to SRD and CAD might also be simultaneously programmed or reprogrammed if mediated by epigenetic marks.

Briefly, summarizing the common brain anatomical structures that participate in CV and behavioral responses, there are at least three instances playing a regulatory role in the functioning of the brain-heart axis. They are the central nervous system (CNS), which is the main instrument to explore and evaluate environmental situations and in which the prefrontal cortex (PFC) plays a central role; the amygdala with its extension in the bed nucleus of the stria terminalis (BNST); the insula and the autonomous nervous system (ANS) with its sympathetic (SNS), parasympathetic, and enteric divisions [34]; and the recently considered intrinsic cardiac nervous system (ICNS) [35]. The PFC forms a binomium with the amygdala in the control of physiological and behavioral responses [34]. The human PFC is unique since it allows for symbolic expression that renders it possible for the individual to evaluate the environmental stimulus and interpret it as it really is, or as a scary or insignificant stimulus that goes beyond the real environmental situation. The response generated to the environmental stimulus may thus correspond to the real situation or to a fantasized one. Furthermore, the PFC is the neuro-anatomo-physiologic substrate that determines decision making with the possibility of contributing to the development of risk of or resilience to diseases or to the reprogramming of the effects of early programming [21,22]. The interpretation and the responses given by the PFC to these conditions also determine the epigenetic marks [32].

In addition to the above mentioned neuronal structures and as mentioned previously, neuroinmunoendocrine mediators have been proposed to jointly participate in the common origin of SRD and CMD, including CAD. The systems responding to stress include a complex network of mediators with complex interactions whose knowledge is continually expanding. The variations in the production of these mediators could be programmed since the early stages of development, leading to the appearance of complex diseases in adulthood or to resilience to them, and could also be reprogrammed. In this paper, we analyze some of the common mediators of SRD and CAD involved in the comorbidity of these diseases and their mechanisms of action, the evidence of their programming in early development, and the possibility to act upon this predisposition and modify the outcome. Although studies on epigenetics of the comorbidity are absent, there are enough data on the epigenetics of each of the molecules participating in these diseases. Therefore, we are putting forward the theoretical hypothesis that epigenetics might contribute to their simultaneous appearance.

## 2. Similar Underlying Neuroendocrine Function in Psychiatric and Cardiometabolic Diseases

Cells from the whole body and those pertaining to neural structures synthesize and release different amounts of chemical molecules, depending on the tissue to which they belong. These molecules mediate communication between cells from the same tissue and with those of other tissues. Communication also depends on the expression of specific receptors that receive the signals. Cells from the heart and brain require similar chemical mediators.

Many neuroactive substances modulate the CV system through their action on the CNS or act directly on the heart and vessels. Some neurotransmitters, including some of the classical ones (acetylcholine, noradrenaline, adrenaline, dopamine, serotonin, and histamine), are among them. Neuropeptides are also included, such as vasopressin, oxytocin, natriuretic peptides (NPs), corticotrophin, angiotensin, thyroxin, Y neuropeptide, leptin, endothelin, orexins, apelins, interleukin 1b, and tumor necrosis factor-α (TNF-α) as well as steroids such as mineralo- and glucocorticoids, estrogens, and testosterone [36]. Other substances involved are purines and gastric system transmitters (nitric oxide, hydrogen sulphide) [36].

The effect of neurotransmitters is usually of a short duration and can be stimulatory or inhibitory. In contrast, the effects of neuropeptides are long lasting and may contribute to the long-term modulation and restructuring of regulatory CV networks, whilst also having relevance in stress situations [36]. Since these neuroactive substances have direct effects upon the CV system and modulate this system through their actions on the CNS, they could have co-joint effects on CAD and SRD. In the following sections, we describe the effects of some of the most important peptides that may have this dual regulation and the evidence of their early programming. The reports on the epigenetic regulation of neuro- and cardio-endocrine mediators possibly having co-joint effects on the risk of developing SRD and CAD are summarized in Table 1.

## 3. Psychiatric and Cardiometabolic Mediators That Could Be under Early Programming Regulation

### 3.1. Hypothalamus-Hypophysis-Adrenal Gland (HHA) Axis Mediators

After a stressful event, the HHA axis is activated. The hypothalamus secretes the corticotrophin releasing factor (CRF) which, in turn, regulates the secretion of the adrenocorticotrophic hormone (ACTH) by the hypophysis. ACTH regulates the secretion by the adrenal cortex of important stress reaction mediators such as the glucocorticoid, cortisol in humans (or corticosterone in rats), and of mineralocorticoids. Although CRF was initially characterized as a regulator of ACTH, it is nowadays known that it participates in the control of the CV and gastrointestinal function, in behavioral and physiological responses to stress, and in the control of food intake [52,53,54,55,56]. The CRF system includes the main CRF molecule and similar peptides known as urocortins 1, 2, and 3. Their effects are mediated by the CRF1 and 2 receptors and by the CRF binding protein [57].

When stress is persistent and severe, the signaling from CRF promotes a decrease in dendritic branching which requires glutamatergic stimulation mediated through *N*-methyl-d-aspartate (NMDA) receptors [58]. ACTH signaling promotes adrenaline secretion by the suprarenal gland, which overstimulates the ANS with a predominance of the sympathetic over the parasympathetic branches, generating an increase in inflammatory cytokines and a decrease of anti-inflammatory mediators. This situation is present in SRD and CMD and other systemic pathological conditions [59,60] and the activation of this axis is linked to an increased risk of developing psychiatric disorders, particularly SRD, and is also related to an elevated risk of CV diseases such as CAD.

**Figure 1:**
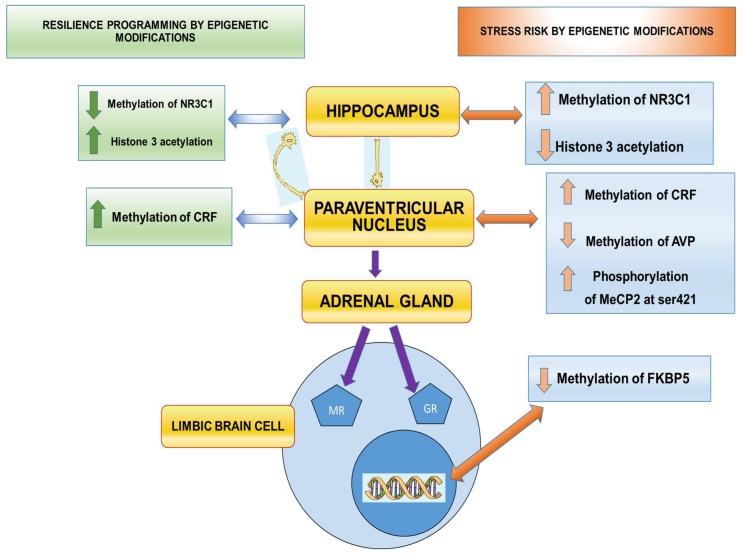
Schematic representation of the epigenetic regulation of the hypothalamus-hypophysis-adrenal axis. The right side of the figure illustrates the epigenetic modifications that lead to stress vulnerability. The left side represents epigenetic variations leading to resilience. NR3C1: steroid receptor gene, pMeCP2: phosphorylated protein related to methylation of histones, CRF; corticotrophin releasing factor gene, AVP: arginine vasopressin gene, FKBP5: gene coding for chaperones for the expression of glucocorticoid receptors (GR) and mineralocorticoid receptors (MR). Purple arrows indicate the hypothalamus-hypophysis-adrenal axis. Blue arrows indicate the epigenetic modifications that lead to resilience at each structure. Brown arrows indicate the epigenetic modifications that lead to risk at each structure.

The HHA axis activity can be modified at different levels, including its basal functioning, its circadian cycles, and/or the degree of response to acute stress. Changes in circadian rhythms, including that of the HHA axis, have been associated with the development of metabolic syndrome and its CV consequences [61]. Although the mechanisms involved in the regulation of this axis remain unknown, there is participation of the density of glucocorticoid and mineralocorticoid receptors in different target regions of the brain such as the hypothalamus or the hippocampus [62]. Changes in the activity of the HHA axis might also be due to variations in the hormone concentrations or to the density of peripheral receptors [63]. Stress exposure activates the HHA-axis and the release of corticosteroids, which activate the glucocorticoid receptors (GR) and the mineralocorticoid receptors (MR) in the brain. The GR mediate stress responses and their effects have been widely studied; however, it has been reported that the MR participate in resilience. Increasing the activity or expression of brain MR may prevent or reverse symptoms of stress-related states and may participate in the prevention and treatment of other psychiatric disorders [64] (Figure 1).

CRF and urocortins might participate in the mediation of a variety of pathophysiological conditions involving abnormal responses to stressors such as AD. Mice over-expressing CRF show anxiogenic-like responses compared to wild-type mice. The CRF-1 receptor may mediate CRF-like neuropeptide effects on the behavioral profile [65]. Results obtained with peptide CRF receptor antagonists in animal models of anxiety demonstrated that the blockade of central CRF receptors may yield anxiolytic-like activity [66]. Furthermore, mice lacking the CRF-1 receptor showed an anxiolytic-like response to stressors. Urocortins have many of the effects similar to those of CRF but are also significantly more potent than CRF. Brain sites for the behavioral effects of CRF include the locus coeruleus, paraventricular nucleus (PVN) of the hypothalamus, the BNST, and the central nucleus of the amygdala. CRF systems in the brain have a unique role in mediating behavioral responses to diverse stressors. These systems may be particularly important in situations where an organism must mobilize not only the pituitary adrenal system, but also the central nervous system, in response to an environmental challenge [65].

CRF and urocortins also have cardioprotective effects that are mediated by cardiac CRF_2_ receptors [67]. Urocortins 1, 2, and 3 are present in the heart, with the abundant expression of urocortins 2 and 3 in myocardium [68]. Urocortins are cardioprotective when added to post-ischemic/hypoxic cardiomyocytes or to the isolated intact heart during reperfusion after regional ischemia [69]. Urocortin 2 and urocortin 3 reduce infarct size in isolated rat hearts after post-ischemia reperfusion [70] and also protect the human heart from reperfusion injury [71]. The cardioprotective effects of urocortins involve many signal transduction pathways which include the protein kinase C (PKC) epsilon isozyme that mediates the effects of urocortins in primary rat cardiomyocytes. Some PKC inhibitors eliminate urocortin 1-induced cardioprotection of an isolated rat heart following ischemia-reperfusion [72]. Urocortin 1, 2, and 3—induced cardioprotection also involves mitogen activated protein (MAP) kinase signaling [70,73]. Cardioprotective effects of urocortins may be dissociable from their hypertrophic effects, which also involve the activation of a phosphatidylinositol-3 (PI-3) kinase/protein kinase B (PKB, also known as Akt)-dependent pathway [73,74].

Furthermore, insulin signaling, in the hypothalamus, whose malfunction constitutes a CV risk factor, regulates the HHA axis after stress through a negative feedback loop. Insulin normally provides an inhibitory tone to the axis and is regulated in a reciprocal manner by corticosterone. Altered signaling through the insulin receptor in the hypothalamus modifies the neuroendocrine response to acute psychological stress. Insulin signaling may promote or inhibit the release of other hormones such as vasopressin, depending on the region to which it binds [75,76].

**Figure 2:**
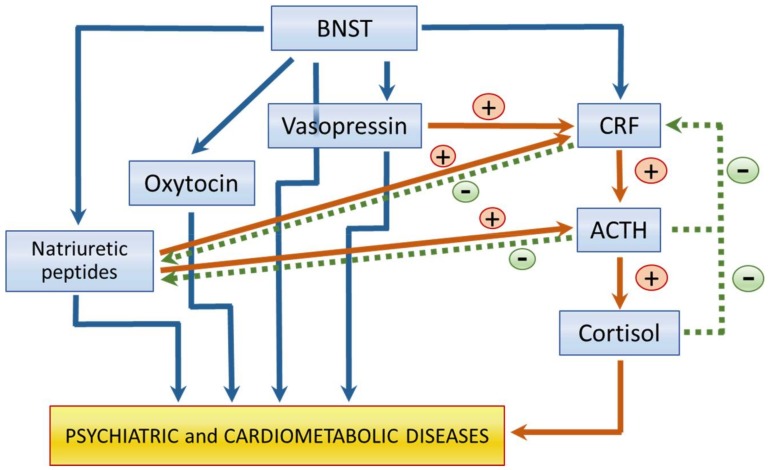
Regulation of neuro- and cardio-active substances by the Bed nucleus of the stria terminalis (BNST) and crosstalk established between them. Blue arrows indicate mediators that are released from the BNST and their relation to psychiatric and cardiometabolic diseases. Brown arrows and + symbol indicate that the pathways increase the mediator release. Green arrows and − symbol indicate that the pathway inhibits the mediator release

The regulation of comorbid cardiac and brain conditions might be the consequence of the anatomical and functional relation between the HHA axis and the BNST (Figure 2). The BNST regulates both psychiatric conditions and heart functioning. The anterolateral and posteromedial subregions of the BNST send projections directly to the PVN of the hypothalamus, in which CRF is stored [77]. Fibers containing CRF are present in the oval and fusiform region of the anterior BNST. The effects of BNST on the stress response depend on the duration of stress. Lesions in the anteroventral BNST reduce the acute response of the HHA axis to stress, but increase the response to chronic stress [78,79]. Lesions in the posteromedial BNST increase the response to chronic stress [78,79] (Figure 2).

The HHA axis constitutes a good example of early programming since fetal exposure to maternal cortisol during development modifies the neuroendocrine system response related to stress and leads to psychopathology in adulthood. There are numerous evidences of early programming of CV disease triggered by changes in early life of the functioning of the HHA axis. High fasting cortisol levels during early development determine low birth weight which is associated with CMD in the adult [27]. Epigenetic signals determine the risk of suffering from stress or the ability to have an adequate level of resilience. Epigenetic marks leading to this regulation include: (1) methylation levels of steroid receptor genes such as *NR3C1* [37,80]. Exposure to stressful events during early stages of life lead to differences in the methylation levels of the genes of *GR* in the hypothalamus and modify the impact of stressful events in adulthood [81]; (2) Acetylation of histones and phosphorylation of proteins related to methylation of histones including pMeCP2 [37,82]; (3) methylation or acetylation of neuroactive mediator genes such as *CRF* or *arginine vasopressin (AVP)* [37] or of chaperones involved in the expression of *GR* and *MR* such as FKBP5 [37,83] (Figure 1).

### 3.2. Oxytocin and Vasopressin

Oxytocin and vasopressin are synthesized by the cells of the PVN and supraoptic nucleus of the hypothalamus and then stored in the posterior hypophysis cells for their subsequent liberation to the circulation [84,85]. These peptides regulate the water balance, the CV function, birth, and lactation [86,87,88,89,90].

Regarding the role of oxytocin and vasopressin in SRD, peripheral [91] or central [92] administration of oxytocin has an anxiolytic-like effect in rats. Oxytocin infused into the central nucleus of the amygdala, but not the ventromedial nucleus of the hypothalamus, was anxiolytic and therefore, the effects are brain region-specific [93]. A specific oxytocin antagonist given centrally enhanced anxiety-related behavior in pregnant and lactating rats, without exerting similar effects in other female or male animals. Thus, the anxiolytic action of central oxytocin is present at a higher degree when the brain oxytocin system is activated. These reproduction-dependent behavioral alterations might be related to the complex pattern of maternal behavior, which includes an increased aggressive behavior in order to protect the offspring [94].

In contrast, vasopressin plays an important role in behavior and psychiatric disorders, exhibiting effects that include the coordination of different central functions such as learning, memory, and emotionality by acting upon the septum. Vasopressin determines behavioral responses to environmental demands. It participates in social recognition, aggression, reproduction, parental behavior, and affiliation [95]. There is evidence of the involvement of septal receptors to vasopressin in the regulation of the anxiety-related behavior of rats. More vasopressin mRNA is present in the PVN in hyper-anxious animals without any difference in oxytocin messenger RNA [96]. Vasopressin is also involved in the regulation of ACTH secretion, together with CRF. In healthy subjects, an elevation of cortisol levels is compensated for by a decrease in CRF and ACTH by a negative feedback circuit. However, ACTH secretion can also be directly stimulated by vasopressin in the hypophysis through the vasopressin-1B receptors. This mechanism leads to a hypersecretion of cortisol. It is important to mention that the vasopressin-induced ACTH hypersecretion, which could be the mechanism used in the BNST, does not have a negative feedback mechanism, and high vasopressin levels might result in a vicious cycle [97] (Figure 2). In addition to its increased expression in the PVN in hyper-anxious animals, more vasopressin is released within the PVN under basal conditions and upon stimulation in freely behaving rats, suggesting that centrally released vasopressin plays a major role in the hyper-reactive HHA axis [98].

Therefore, vasopressin might participate in the development of stress and depression, while oxytocin might favor resilience. Oxytocin and vasopressin also have opposite effects upon CV control. Vasopressin is a potent vasoconstrictor that increases blood pressure, while oxytocin causes vasodilation [89,99,100,101,102]. In relation to CAD, continuous in vivo oxytocin delivery improves the cardiac healing process in rats, as well as cardiac work, reduces inflammation, and stimulates angiogenesis. Oxytocin has well-known CV activities which include: lowering blood pressure, negative cardiac inotropy and chronotropy, parasympathetic neuromodulation, vasodilatation, and anti-inflammatory, antioxidative, and metabolic effects. In addition, it also has the capacity to generate cardiomyocytes from various types of stem cells, including the cardiac side population. Mesenchymal cells preconditioned with oxytocin are resistant to apoptosis and express endothelial cell markers. Ischemia/reperfusion, by decreasing oxytocin receptor expression in the heart, may result in vasopressin-inducing vasoconstriction and cardiac dysfunction in the injured heart. In pathological conditions, oxytocin exerts anti-inflammatory and cardioprotective properties, and improves vascular and metabolic functions, and these outcomes are mediated, at least in part, by stimulating cardioprotective mediators, such as nitric oxide and atrial natriuretic peptide (ANP) [87]. The oxytocin effects are lost in rats having post stroke cardiac failure [103].

In turn, vasopressin has dose-dependent effects on both cardiac contractility and coronary arterial tone. Low-dose vasopressin infusion has no significant CV effect in normal mice; however, vasopressin infusion depresses the myocardial function in ischemia/reperfusion models and increases mortality in comparison with both saline and dobutamine treated animals. Following ischemic injury to the myocardium, vasopressin exerts a strong negative inotropic effect on the heart, leading to a decline in cardiac ejection fraction. This decline is not mediated through changes in left ventricular preload or afterload and therefore, a direct cardiac effect is possible [104]. High levels of circulating vasopressin, determined by levels of copeptin, are a new risk factor for the development of diabetes, metabolic syndrome, and CV morbi-mortality [97]. The most probable cause for this relation is an insufficient water intake that leads to an elevated secretion of vasopressin, which in turn increases the secretion of ACTH and cortisol.

Oxytocin and vasopressin might participate in the simultaneous comorbidity of SRD and CMD, including SRD and CAD, through the effect of BNST, an anatomic structure that regulates both mood and CV function, on their secretion. Vasopressin release to the circulation mediates pressure responses that follow the application of noradrenaline, carbachol, and gamma amino butyric acid (GABA) to the BNST [105,106] and are thus related to CV control by the brain. There are also massive projections from the BNST to magnocellular neurons in the paraventricular and supraoptic nucleuses [77,107] which produce vasopressin and oxytocin and that are the final components of the neuronal pathways that regulate a great variety of neuroendocrine functions (Figure 2).

Regarding early programming of oxytocin and vasopressin, it has been reported that the elevated estrogen levels during pregnancy modify the oxytocin receptors in the limbic system, reducing maternal fear to new stimuli, thus rendering the mother more receptive to social signals from the newborn, which favor a better environment for child development [39]. Changes in the maternal mood might affect the product. Oxytocin levels during early life determine the risk of developing CMD during adulthood [40]. 

The oxytocin and vasopressin neuroendocrine systems are involved in sensibility adaptations to stress and their functioning during adulthood might be programmed from the early stages of life. Postnatal chronic stress induces a behavior similar to that of depression when offspring reach adulthood and stimulates the expression of the vasopressin receptor-1 in the hippocampus in the young adult stage. Furthermore, when two sources of stress are combined during early life, the expression of the oxytocin gene receptor is increased in the hippocampus when the individuals reach adulthood [41]. Regarding the development of early programmed CV diseases, maternal water restriction induces the risk of developing hypertension in the offspring when they reach adulthood, probably by alterations in the vasopressin receptors [42].

### 3.3. Natriuretic Peptides (NPs)

NPs are compounds that act in an endocrine or paracrine way to regulate extracellular liquid volume and arterial pressure through stimulation of the sodium excretion by the kidney. Peripheral atrial natriuretic peptides (ANP) and their bioactive fragments, together with their corresponding receptors (natriuretic peptide receptors NPR-A, NPR-B, and NPR-C), are involved in diuresis and blood pressure regulation. NPRs are also found in the brain. NPR-A and NPR-C are found on neurons and astrocytes, while NPR-B is mainly located on neurons and partially co-localizes with NPR-A. In the CNS of man and rodents, NPR-A is mainly found in the cortex and hippocampus, whereas NPR-B is present in the amygdala and several brainstem regulatory sites. NPR-C is widely found within the CNS, i.e., in neocortex, limbic cortex, the hippocampal area, and the amygdala. Besides the important cardio-renal function of NPs, they also have vasorelaxant effects and they inhibit the renin–angiotensin-aldosterone system (RAAS) and the sympathetic nervous system (SNS) [108,109]. Circulating ANP and vasopressin can interact to attenuate the central pressor effects of vasopressin [36,110,111,112,113,114], and brain natriuretic peptide (BNP) suppresses the secretion of vasopressin [104]. All of these brain regions participate in the regulation of mood and CV responses.

NPs preserve vascular health in both endothelial and vascular smooth muscle cells by interfering with the key mechanisms that promote atherosclerosis including inflammation, thus participating in CAD [108,109]. Moreover, BNP increases in a broad range of diseases including myocardial ischemia, ventricular remodeling, and overload. In addition, NPs are known to exert anti-hypertrophic and anti-fibrotic roles within the heart [106,107]. In the control of NPs release, mechanical factors such as increased volume overload and myocyte stress play an important role, but hormonal stimuli are also involved [108,109,115,116,117,118,119]. As a result of their complex CV properties, NPs are currently viewed as active players in the process of CV remodeling and in the natural history of heart failure. ANP can inhibit early activation of neurohormonal factors and inflammation after reperfusion therapy. It can also reverse arrhythmias, apoptosis of cardiac myocytes and endothelial cells, and limit infarct size and left ventricular remodeling, thereby improving left ventricular function in animal models with ischemia/reperfusion injury [120]. These peptides also modulate glucose uptake through the Glut4 receptor [121].

NPs also participate in numerous brain functions. They are found throughout the CNS, where they represent an important neuromodulatory system regulating emotional behavior, such as anxiety and arousal, and the consequences of stress hormone release and ANS activation. NPs are specifically involved in the regulation of the HHA system. The effects of ANP seem to be mediated through the inhibition of the HHA axis by decreasing the liberation of CRF and ACTH, which in turn stimulate the liberation of ANP, establishing a feedback circuit. In man and rodents, ANP inhibits the HHA system at all regulatory levels and reduces anxiety levels. The central or peripheral administration of ANP diminishes anxiety-related behavior in rats [122]. In healthy individuals, a pretreatment with ANP partially blocked the sympathetic response induced by an injection of CRF [123]. Moreover, panic disorder and concomitant ACTH and cortisol secretion elicited by stimulation with cholecystokinin-tetrapeptide were also attenuated by ANP infusions in patients, as well as in healthy volunteers. Patients having anxiety-related disorders such as panic [124] and post-traumatic stress [125] have decreased levels of ANP and its secretion is faster and more pronounced during experimentally induced panic attacks. Hence, in man and rodents, ANP reduces anxiety and terminates panic attacks and their neuroendocrine consequences. The inhibitory potency of ANP could explain the unexpected and so far, unresolved failure of autonomic and HPA system activation. Elevated levels of ANP are associated with a lower anxiety level in patients recuperating from cardiac failure [126].

Similar to ANP, BNP also has anxiolytic effects [127]. By contrast, C-type natriuretic (CNP) stimulates cortisol secretion [128] and has an anxiogenic effect in rodents and humans [129,130]. In humans, pretreatment with CNP increased the effect of the anxiogenic agent cholecystokinin 4 [131]. CNP also stimulates the liberation of cortisol and prolactin [132]. 

Immunocytochemistry studies have found NP synthesizing neurons in the BNST, which regulates both CV function and emotions [133] (Figure 2). Therefore, these peptides are present in areas that simultaneously participate in the regulation of CV and mood regulation and could participate in the comorbidity of SRD and CAD.

Among the evidence suggesting that the secretion of NPs is under programming since early stages is the finding of epigenetic modifications in the patterns of NPs secretion between the right and left ventricles and of other proteins that determine the difference in the force of contraction that ventricles exert [43]. There is also hypermethylation of the *BNP* gene in the heart of subjects with rheumatic diseases [134]. There is also a re-activation of specific heart genes including the *ANP* and *BNP* genes at the beginning of the development of heart failure [135].

### 3.4. Renin-Angiotensin-Aldosterone System (RAAS)

The renin-angiotensin system (RAAS) is a circulating and tissue-based hormonal system with a pathway that can be divided into the classic RAAS and the non-classic RAAS. Both pathways play a key role in CV regulation, but are also involved in neurological diseases through peripheral and/or central control systems [136]. The RAAS is an important mediator of stress responses and related pathologies. The RAAS participates in the response to chronic stress and its psychological, CV, and metabolic consequences. A population of angiotensin-sensitive neurons is in charge of the coordination of stress responses and its response is mediated by angiotensin type-1a receptors located in the parvocellular neurosecretory neurons of the PVN of the hypothalamus. These neurons are mainly glutamatergic and project to the exterior portion of the median eminence. These neurons secrete CRF or thyrotropin-releasing hormone, but do not express vasopressin or oxytocin. Stimulation of these neurons promotes the activation of the HHA axis and cause elevations of systolic blood pressure. When these neurons are inhibited, the activity of the axis is diminished, and an anxiety-like behavior is present [137].

The RAAS plays an important role in blood pressure control and volume homeostasis. Inappropriate activation of the RAAS has been implicated in the pathogenesis of hypertension and related CV diseases [138]. In addition to the role of RAAS in hypertension, angiotensin II (Ang II) has been involved in the development of vascular and cardiac hypertrophy and remodeling. It participates in mechanisms that contribute to vascular damage and atherosclerosis. However, the mechanisms responsible for these pathologic effects of Ang II are still unknown since they often occur in the absence of any perturbation of the circulating RAAS, but the dysregulation of some component(s), such as angiotensin converting enzyme (ACE) levels, the balance of angiotensin-receptor subtypes, or even local synthesis of renin or angiotensinogen, could account for them [139]. Recurrent stroke is a major public health concern. Modulation of the RAAS has proven effective in reducing recurrent cardiac events; however, its role in preventing recurrent cerebrovascular events remains unclear [140].

Regarding the regulation of comorbidity of cardiometabolic and neuropsychiatric disorders, angiotensin peptides play an important role in direct CV regulation and are also involved in the central regulation of the CV system. All of the components of the RAS and their receptors are present in the brain and particularly in structures involved in the regulation of the CV system [141]. Ang II receptors are stimulated by angiotensin liberated by neurons located in the brain or by the peptide that arrives via the circulation. Vasopressin and Ang II interact in the central regulation of the resting blood pressure levels and in responses to stress [103]. Furthermore, angiotensin-(1–7) has anxiolytic- and antidepressant-like effects in hypertensive transgenic rats [142].

The secretion levels of the RAAS are subjected to early programming. The abolishment or improper activation of the RAAS may unleash an increase in oxidative stress and in the production of endothelin, which are implied in the development of hypertension, whose origin is linked to the fetal stage [44].

### 3.5. Neuregulins (NRGs)

NRGs comprise a family formed of four members of signaling molecules (NRG1, NRG2, NRG3, and NRG4) that are similar in structure to the epithelial growth factor (EGF). NRGs participate in the communication between cells during development and in diseases. NRGs transmit their signals to target cells through their interaction with tyrosine kinase activity receptors pertaining to EGF, the ErbB family [143]. This family of receptors includes four members: ErbB1, ErbB2, ErbB3, and ErbB4, which activate signaling cascades leading to cell proliferation, migration, differentiation, and survival or apoptosis [143].

Of the NRGs, NRG1 is the most studied and it plays an important role in the early development of the nervous system, heart, and breast gland [46]. In the nervous system, NRG1 binds directly to ErbB3 and ErbB4 receptors and participates in the development of the neural crests and their derivatives such as Schwann cells and SNS neurons. The precursors of Schwann cells and of cranial ganglia are not normally developed in the −/− embryos for NRG1, in which this molecule acts in a paracrine way [45]. In the heart, NRG1 controls the trabeculation of the myocardial musculature. NRG1 and NRG2, together with their receptors ErbB2 y ErbB4, are essential for normal heart development and mediate hypertrophic growth, promoting the survival of embryonal ventricular myocytes [46]. The ErbB family of receptors in endothelial cells induces angiogenesis [144]. Furthermore, homozygous −/− embryos for NRG1 die during embryogenesis and show heart malformations [46].

Type II NRG1 is expressed in the neurocircuitry involved in regulating the HHA axis response to stressors, including the hypothalamic PVN, which integrates and controls the neuroendocrine responses to stress [145,146], thus participating in AD. Gene-environment interactions between *NRG1* and stress in adult humans have been reported [145,146]. When the signaling form NRG1-ErbB4 in the BNST is stopped in mice, anxiogenic responses are produced and neuregulin regulates the presynaptic liberation of GABA [147]. NRG1-ErbB4 signaling is critical to maintaining the GABAergic activity in the amygdala and modulating anxiety-related behavior [148]. Moreover, using a rat model of disrupted Type II NRG1, the hypomorphic *Nrg1^Tn^* rats, it was found that male *Nrg1^Tn^* rats have significantly higher basal corticosterone levels, while female *Nrg1^Tn^* rats show enhanced suppression of corticosteroid secretion after recovery from acute restraint stress [143]. In addition, sex-specific changes in GR and MR expression were found, leading to a disrupted MR/GR balance in the hippocampus of males and amygdala of females [143]. These findings implicate Type II NRG1 participates in stress regulation. Behaviorally, male *Nrg1^Tn^* rats may be more sensitive to changes in their environment, while female rats are less impacted by mildly stressful aspects in their environment [143].

Regarding the participation of neuregulins in CAD, endothelial cells produce neuregulins. The activation of ErbB by NRG can also protect from the ischaemic damage of heart tissue in vivo [149,150] and it can induce angiogenesis [145,150]. The role of NRG-ErbB during arteriogenesis was proven in an endothelial selective NRG knockout model. NRG has a role in adhesion protein receptor integrin, which is needed for the proliferation, migration, and differentiation of endothelial cells during angiogenesis, thus participating in vascular growth and flow recovery, reducing ischemic damage [145,150]. The NRG1/ErbB signaling plays an important role in the maintenance of the adult circadian function that is altered in diabetic cardiomyopathy, which is one of the most important causes of the increased morbi-mortality in diabetic patients [151]. Circulating concentrations of NRG4 are inversely associated with subclinical atherosclerosis in obese adults [152]. This neuregulin participates in the browning of adipose tissue. The NRG1/ErbB signaling also participates in a non-adaptive response in the cardiac dysfunction induced by chronic stress or by an excess of glucocorticoids, conditions which are present during the development of depression and cardiac disease [153,154,155].

Evidence of a possible programming by neuregulins during the early stages of risk/resilience to diseases has been found. Alterations in the peripheral signals from NRG1 during neural development also modify the behavioral traits, inducing their early programming [45]. The development of the dopaminergic system is very vulnerable to circulating levels of ErbB receptor ligands during the pre- and perinatal stages. Once the alterations in the dopaminergic system are established, they persist until after adolescence, being irreversible and following a typical pattern of early programming [156].

The peripheral administration of NRG1 increases the ErbB4 levels in the brain of neonatal mice and elevates the expression, phosphorylation, and enzymatic activity of tyrosine hydroxylases, which increase dopamine levels. The hyperdopaminergic state is maintained until after adolescence in the medial PFC. Therefore, NRG1 neurotrophically contributes to an altered dopaminergic system and to the posterior pathogenesis of neuropsychiatric diseases [45]. Furthermore, altered expression of NRG3 at different stages of development contributes to neurological deficiencies [157].

### 3.6. Purinergic Neuromediators

Purinergic mediators include molecules such as adenine, adenosine triphosphate (ATP), diadenosine polyphosphates (ApnA adenosine), β-nicotinamide adenine dinucleotide (NAD^+^), and ADPribose (ADPR). It is difficult to identify which of these molecules is participating in each case as a mediator since purines are present in all types of cells and are generated by a great variety of sources and liberated by numerous pathways [158].

Adenine compounds participate in neuronal functioning and in neurotransmission. Purine receptors have been identified in plasma membranes and the specific actions of extracellular purines have been reported in different types of cells and tissues. Possible mechanisms of transduction and action have also been identified, as well as destruction pathways and their participation in complex processes including cell motility, proliferation, survival, membrane trafficking, and cell to cell communication. Therefore, nowadays, they are considered as true neurotransmitters [159].

There are a great variety of purine receptors, which have been classified as receptors for P1 and nucleotide receptors P2. P1 receptors are linked to G proteins and comprise the A1, A2A, A2B, and A3 subtypes. The A1 and A3 receptors are coupled to the Gi/o family of G proteins which inhibit the cyclic AMP production, while A2A and A2B receptors stimulate cyclic adenosine mono phosphate (AMP) production through the Gs protein. P2 receptors are subdivided into the seven ionotropic P2X receptors (P2X1-7) and the eight metabotropic P2Y receptors (P2Y1, 2, 4, 6, 11–14). The P2X receptors are classical ionic channels whose opening is ligand-dependent and are permeable to ions such as sodium (Na^+^), potassium (K^+^), and calcium (Ca^2+^). The P2Y receptors are linked to G proteins which activate the phospholipase C/inositol triphosphate intracellular Ca^2+^ liberating pathway [158].

ATP and adenosine are the main purines acting as excitatory and inhibitor neurotransmitters in the nervous system, respectively. The purinergic signaling could explain how the neuronal activity is associated with energetic changes and energy homeostasis, particularly in mental disorders. Purinergic signaling may play an important role in regulating synaptic activity and promoting normal neuroadaptation. Therefore, a dysfunctional mitochondrial structure, abnormal subcellular location, function, and an inadequate energy and ATP management could alter purinergic signaling. This type of functional alteration in the CNS functioning might be associated with aberrant behavior [160]. Adenosine plays a role in SRD. Adenosine receptors are especially abundant in the CNS. Purinergic signaling via the P2X7 receptor is present when microglia are activated by repeated stress and they can trigger prostaglandin E2 (PGE2) and interleukin 1β (IL-1β) production. Microglia act as a source of inflammation-related molecules which have been linked to medial PFC dysfunction. Prolonged or intensive stress results in emotional and cognitive deficits and is a major risk factor for psychiatric disorders, including anxiety [161].

Adenosine also plays a role in CAD. This purine is released by ischemic tissue and its concentration jumps 100-fold during periods of oxygen depletion and ischemia [162]. Adenosine receptors are present in the heart in smaller numbers than in the brain. The A(1)-receptors are located on cardiomyocytes and vascular smooth muscle cells, A(2)-receptors on endothelial and vascular smooth muscle cells, and A(3)-receptors on ventricular myocytes [162]. Adenosine constitutes a key trigger of ischemic preconditioning [163]. Ischemic preconditioning by endogenous adenosine takes place through A(1)- and A(3)-receptors. A(2A/B)-receptor activation results in vasodilation. Adenosine receptors play an important role in preconditioning during coronary angioplasty [164,165,166].

ATP also participates in heart control as a co transmitter of the sympathetic, parasympathetic, sensory-motor nerve, and intracardiac neurons. Adenosine nucleotides and nucleosides act on purinergic receptors in cardiomyocytes of the sino-atrial and atrio-ventricular nodes, on cardiac fibroblasts, and in cells of the coronary vessels. The vascular tone is controlled by a dual mechanism in which the ATP liberated from the perivascular sympathetic nerves causes P2X1 receptor vasoconstriction. Endothelial cells, in turn, also liberate ATP in response to changes in blood flow (through shear stress) or to hypoxia acting upon P2 receptors of the same endothelial cells to produce nitric oxide, endothelial derived hyperpolarizing factor, or prostaglandins to cause vasodilation [167].

With respect to early programming, there are very few molecules with a higher potential of having an influence on mammalian development than adenine nucleosides. Adenosine levels rapidly increase under hypoxic or inflammatory conditions. When pregnant mice are treated with an adenosine antagonist such as methylxanthines (caffeine or theophylline) during embryogenesis, upon reaching adulthood, pretreated mice show abnormal cardiac function. Adenosine acts through A1ARs receptors in the protection of embryos against intrauterine stress [48]. These receptors are the mediators of the long-term effects of the in utero exposition to caffeine and their later cardiac effects and have been linked to epigenetic changes mediated by DNA methylation [49].

There are also a growing number of studies showing evidence that the nucleotide signaling plays an important role in the development of the nervous system. Nucleotide signaling is associated with progenitor cell proliferation, cellular migration, interaction and differentiation of neurons and glia, and in the formation of synaptic nets. Nucleotide receptors might play a relevant role in the epigenetic regulation of genes [47].

### 3.7. Inflammatory Mediators

Inflammation is a physiopathologic process accompanying depression, anxiety, and CV diseases [168,169]. Diet might simultaneously program CMD and the brain through inflammatory mechanisms. Obesity and lipid rich diets are independently associated with excessive systemic levels of inflammatory mediators [170]. Atherogenesis is induced by inflammatory damage of endothelial cells and immune cells, mainly T and B cells, and monocytes and macrophages are involved in its development. Inflammatory cells accumulate in the inner membrane of the artery leading to a local inflammatory process due to the secretion of reactive oxygen species, inflammatory cytokines, and metalloproteinases. These molecules accelerate the development of atherosclerotic lesions in the arteries. Chronic inflammation may generate endothelial dysfunction that results in a decrease in the concentration of elastin and collagen and to increased apoptosis of smooth muscle cells of the intima. The integrity of the fibrous cap that covers a layer of thrombogenic plaque from contact with blood elements is reduced. Chronic inflammation promotes the formation of a necrotic core, composed of dead smooth muscle cells, macrophages, and foam cells formed by the phagocytosis of oxidized lipid molecules. Plaque rupture and thrombus formation within the coronary artery are a consequence of the thin fibrous cap and a large necrotic core [168]. Inflammation is also present in metabolic disorders such as metabolic syndrome and obesity, which favor systemic atherosclerosis [170] and focalized atherosclerosis in specific regions such as the carotid arteries, with the consequent appearance of neurological events [171].

Also, important brain functions such as neurotransmitter metabolism, neuroendocrine function, synaptic plasticity, and the neural circuitry of mood are regulated by cytokine signaling in the brain. Dysregulation in cytokine signaling might lead to anxiety and cognitive dysfunction. There is a bidirectional interaction between the immune system and the CNS in which psychological stress modulates cytokine production and cytokines determine psychological stress, and its presence is one of the most relevant findings in the last decades. The role of neuroimmunology in anxiety has been studied and oxidative stress has been found to work as a sensor of distress leading to neuroinflammation, thus participating in anxiety disorders [168]. In physically healthy subjects, there is a positive correlation with C-reactive protein (PCR), tumoral necrosis factor-alpha (TNF-α), interleukin (IL) 6, homocysteine, and fibrinogen, and the severity of the anxious symptoms in both genders, except for TNFα for females [172]. Additionally, anxiety overstimulates the sympathetic branch of the ANS. A number of studies have found autonomic changes associated with generalized anxiety disorder and worry. In one of them, which focused on over anxious humans, heart rate was elevated and respiratory sinus arrhythmia (a measure of vagal tone) was reduced at baseline [173,174]. Subjects that exhibit anxiety show a failure to reduce sympathetic tone [175]. Furthermore, the chronic activation of the sympathetic system has recently been proposed to have pro inflammatory effects [59,60].

The CV and nervous systems share a large group of mediators, among which cytokines and their receptors are counted. Thus, the altered expression of these molecules in any of these systems influences the other in a reciprocal manner. Among the inflammatory mediators that may play a role in the co joint appearance of CAD and SRD are IL-6, TNFα, soluble cell adhesion molecule 1, and acute phase proteins, particularly C-reactive protein (PCR), as well as a decrease in the anti-inflammatory cytokines IL-4 and IL-10. The cytokine profile that underlies the psychiatric disorder present in patients showing acute post stroke anxiety might account for the increased risk condition to develop new CV events. In chronic inflammatory diseases (such as multiple sclerosis), the permeability of the hematoencephalic barrier is disrupted, allowing for the flow of cytokines and other proinflammatory molecules [175,176]. This entry of cytokines could directly promote brain inflammation.

Regarding early programming, for more than a decade, it has been considered that inflammation is one of the mechanisms that alters the structure of chromatin through signaling pathways activated by necrosis factor-κB (NF-κB) [177]. These pathways modify histone methylation patterns and genic expression [178]. Inflammation is a common pathway of stress-related diseases and plays a favorable role in tissue remodeling [179]. Due to the dual role of immunological mediators such as interleukin 6 and 1β (IL-6, IL-1β) in placental function and brain development, any disorganization in the balance of growth factors or neurotransmitters, such as serotonin, caused by premature infections, may alter the development of the fetal brain in a permanent way and predispose to CV events [50,51]. Changes in maternal diet could also participate in this process.

## 4. Possible Re-Programming of Expression of Cardio and Neuroactive Substances Participating in the Comorbidity of Neuropsychiatric and Cardiometabolic Diseases

At present, epigenetics is considered as a novel area for new therapies [180,181,182,183,184] that may act by returning to chromatin to its previous state before being remodeled by environmental factors and reverse histone modifications that may be contributing to the development of abnormal phenotypes associated with diseases including SRD and CAD [21]. These therapies include lifestyle changes that involve decision making and therefore participation of the PFC, currently used medications, and newly developed ones.

In contrast to genetic mutations, the plasticity of epigenetic changes makes them attractive candidates for prevention or reversion by non-pharmacological intervention. The free will of the individuals to undertake healthy eating habits, perform exercise, and increase mental activity might help induce epigenetic modifications. Dietary lifestyles may alter epigenetic cues in neuropsychiatric and CMD risk in utero, after birth, or during life, since they contribute to the risk of developing these diseases by metabolic re programming of the epigenome [185].

Several studies have reported that intestinal microbial steady-state imbalances can cause a range of metabolic diseases [186,187,188]. Also, the influence of gut microbioma on microbe-related diseases in neuropsychiatric subjects has been explored in a number of studies. In 2004, Sudo et al. [189] were the first to show that bacteria in the gut can influence stress responses. The precise mechanisms for gut-brain interaction remain unknown. Recently, in a study with maternally separated mice, it was suggested that disruptions to the normal development of the gut microbiome may influence future physical and mental health of the offspring [190]. Elsewhere, Hoban et al. 2017 [191] reported that the germ-free mice had blunted fear responses. In 2013, Tillisch et al. [192] administered probiotic yogurt to a group of healthy humans and found that they had a reduced brain response to negative images.

This gut-brain connection could have clinical implications, as influencing the gut microbiome through diet may serve to ameliorate some psychiatric disorders. Dinan et al. coined the term “psychobiotics” in 2013 [193] to describe live organisms that, when ingested, produce health benefits in patients with psychiatric illness. These include foods containing probiotics, live strains of gut-friendly bacteria.

Dietary interventions using natural compounds have been proposed to modify epigenetic cues. A growing number of epidemiologic studies point to a link between the ingestion of nutritional polyphenols and health benefits [194]. A mixture of resveratrol and quercetin has proven to be effective to revert many of the altered variables caused by metabolic syndrome by regulating the histone deacetylase activity of sirtuins [195].

Other interventions proposed include the reduction of glutamatergic receptor activation. This intervention has been found to be enough to counteract the negative effects of increased maternal care on increased CRF signaling [196]. This effect requires enhanced nuclear levels and recruitment of the transcriptional repressor neuron restrictive silencing factor (NRSF), also known as repressor element-1 silencing transcription factor, to the *Crh* gene [197,198]. NRSF chromatin binding was accompanied by methylation in CpG binding protein 2 (MeCP2) sites and was followed by accumulation of repressive epigenetic marks in the hypothalamus of immature and adult rats that had experienced increased maternal care [196]. These mechanisms provide a new mechanistic pathway from early-life experience to phenotypic effects that result in human health and disease.

Another pharmacological manipulation in the adult that was able to reverse some of the effects of environmental influences such as maternal behavior and diet that caused epigenetic changes in neurons by altering histone acetylation, DNA methylation, and nerve growth factor-inducible protein A transcription factor binding, thus inducing long-term changes in *GR* gene expression, was Trichostatin A and L-methionine administration. They influenced the epigenetic status of critical loci in the brain [199].

Perinatal exposure to endocrine disrupting compounds such as xenoestrogens increases the risk of diseases by (re)programming the epigenome via alterations in DNA and histone methylation. Xenoestrogens induce nongenomic signaling to activate PI3K/AKT, resulting in AKT phosphorylation and inactivation of the histone methyltransferase, thus providing a direct link to disruption of the epigenome [22]. Thus, inhibition of this pathway in a selective manner could also lead to re programming in diseases.

Several drugs with a potential capacity to modulate the epigenetic machinery and that could be suggested as new drugs for the treatment of several diseases have been proposed [200,201,202,203,204,205,206,207]. These drugs have the capacity to modulate the activity of histone modifying enzymes that are related to DNA methylation and could therefore rescue the normal conformational structure of the chromatin and revert the changes induced by alterations in the environment. They could slow, stop, or even revert the long-term effects that increase the risk for these diseases [208,209,210].

The area of study of epigenetic therapies is still in its beginnings and new and effective treatments will follow in the near future as experimental data accumulates and is tested for human use. Furthermore, the response of the epigenome to environmental insults throughout life seems not to be an accidental aberration happening only in early life and always leading to pathology, but a biological mechanism that helps adaptability of the genome to altered environments during life. It would be important to delineate if only chemical exposures such as diet or drugs or toxins can affect the epigenome or if the social environment can also act on it. This would imply that there might be signaling pathways which link extracellular environmental exposures and epigenetic machineries in mature somatic cells and that correct environmental exposures, including changes in the social environment, which might reverse damaging signals. The prospect that the social environment or individual behavior might alter our genome by modifying the epigenome might provide an explanation for the relationship between socioeconomic status and physical health. Hence new therapies that modify the social environment might be possible and are an intriguing possibility [211].

## 5. Conclusions

Neuro-cardio-immune-endocrine mediators that participate in the comorbidity of CAD and SRD can be subject to early programming and later reprograming. The synthesis, release, and/or expression of receptors of these mediators may be the subject of epigenetic marks. Although there are no studies on the epigenetics of the comorbidity, there are enough data on each of the epigenetics of the molecules participating in these diseases. Therefore, here we put forward the hypothesis that epigenetics might contribute to their comorbidity. At present, more studies have been done on the programming of CMD than of SRD. The study of possible re programming therapies is an area of opportunity for the treatment of neuropsychiatric and CV diseases offering great relevance. The knowledge of the mechanisms underlying CMD and SRD is important for decision making to safeguard health. This could favor comprehensive health guidelines including actions that influence not only the brain-heart axis parameters, but the entire economy of the organism.

## Figures and Tables

**Table 1:** Evidence of early programming of psychiatric and cardiometabolic diseases by neuro- and cardio-active substances.

Neuro-and Cardio-Active Substance	Early Programming of Psychiatric Disorders	Early Programming of Cardiometabolic Diseases
Cortisol	Changes in concentration during early stages determine the risk to suffer stress or have an adequate level of resilience [37].	High fasting levels during early development determine low birthweight which is associated with CMD [27,38].
Oxytocin	Elevated estrogen levels during pregnancy determine oxytocin receptors in the limbic system which determine the risk of psychiatric disorders [39].	Oxytocin levels during early life determine the risk of CMD in the adult [40].
Vasopressin	Expression of vasopressin receptors is determined by stress in the early postnatal stages and induces a behavior similar to depression [41].	Maternal water restriction induces risk of hypertension in the offspring when they reach adulthood, probably by alterations in vasopressin receptors [42].
Natriuretic peptides		There are epigenetic modifications in the pattern of secretion of these peptides and of other proteins that determine the force of ventricular contraction throughout life [43].
Renin-angiotensin system		The improper activation of the renin-angiotensin-aldosterone system (RAAS) from early stages is implied in the development of hypertension linked to the fetal stage [44].
Neuregulins	Alterations of neuregulin 1 during neural development modify behavioral traits. This contributes to a hyperdopaminergic trait and to the pathogenesis of schizophrenia [45].	Neuregulin 1 is essential for normal heart development, promoting survival of embryonary ventricular myocytes [46].
Purinergic mediators	Adenine nucleotide signaling has an important role in progenitor cell proliferation, cell migration, interaction and differentiation of neurons and glia, and in the formation of synaptic nets during embryogenesis and may alter adult nervous system functioning in the adult [47].	There is abnormal cardiac function in adult offspring of pregnant mice treated with adenosine antagonists [48,49].
Inflammatory mediators	Premature infections cause alterations in the balance of neurotransmitters such as serotonin and may alter in a premature way the development of the fetal brain, having consequences in the adult [50,51].	Cardiometabolic programming might be mediated by inflammatory mediators present during early development [50,51].

## Abbreviations

ACE	angiotensin converting enzyme
ACTH	adrenocorticotrophic hormone
ADPR	ADP ribose
AMP	adenosine mono phosphate
Ang II	angiotensin II
ANP	atrial natriuretic peptide
ANS	autonomous nervous system
ATP	adenosine triphosphate
AVP	arginine vasopressin gene,
AvpR1	vasopressin receptor
BNP	brain natriuretic peptide
BNST	bed nucleus of the stria terminalis
Ca^2+^	calcium
CAD	coronary artery disease
CMD	cardiometabolic diseases
CNP	C-type natriuretic
CNS	central nervous system
CpG	cytosine-phosphate-guanine
CRF	corticotrophin releasing factor
CV	cardiovascular
DNA	deoxyribonucleic acid
EGF	epithelial growth factor
ErbB receptors	receptors pertaining to EGF
FKBP5	gene coding for chaperones
GABA	gamma amino butyric acid
GR	glucocorticoid receptors
HAT	histone acetyltransferases
HDAC	histone deacetylases
HHA	hypothalamus-hypophysis-adrenal
HMT	histone methyltransferases
IL	interleukin
ICNS	intrinsic cardiac nervous system
K^+^	potassium
MAP	mitogen activated protein
MR	mineralocorticoid receptors
mRNA	micro ribonucleic acid
Na^+^	sodium
NAD^+^	β-nicotinamide adenine dinucleotide
NMDA	*N*-methyl-d-aspartate
NPR-A, NPR-B and NPR-C	natriuretic peptide receptors
NPs	natriuretic peptides
NR3C1	steroid receptor gene
NRGs	neuregulins
NRSF	neuron restrictive silencing factor
PFC	prefrontal cortex
PI-3	phosphatidylinositol-3
PKC	protein kinase C
pMeCP2	phosphorylated protein related to methylation of histones
PVN	paraventricular nucleus
RAAS	renin–angiotensin–aldosterone system
RNA	ribonucleic acid
ROS	reactive oxygen species
SNS	sympathetic nervous system
SRD	stress related disorders
TNF-α	tumor necrosis factor alpha
